# Psychosocial status and risk perception among Iranian healthcare workers during the fifth wave of the COVID-19 pandemic

**DOI:** 10.1186/s12960-023-00862-0

**Published:** 2023-09-18

**Authors:** Maryam Khazaee-Pool, Masoud Moradi, Tahereh Pashaei, Koen Ponnet

**Affiliations:** 1 Department of Health Education and Promotion, School of Health, Health Sciences Research Center, Mazandaran University of Medical Sciences, Sari, Iran; 2 Department of Biostatistics and Epidemiology, School of Health, Health Sciences Research Center, Mazandaran University of Medical Sciences, Sari, Iran; 3 Social Determinants of Health Research Center, Research Institute for Health Development, Kurdistan University of Medical Sciences, Sanandaj, Iran; 4 Department of Communication Sciences, imec-mict-Ghent University, Ghent, Belgium

**Keywords:** Healthcare workers, Psychosocial status, COVID-19 pandemic, Risk perception

## Abstract

**Background:**

Healthcare workers (HCWs) are essential resources, and their health and wellbeing are key not only for offering constant and useful care facilities to clients, but also for maintaining the safety of the workforce and patients. The risk of severe mental health problems among HCWs may have increased during large outbreaks of COVID-19. To evaluate the psychosocial status and risk perception of HCWs who participated in treating COVID-19 patients in Northern Iran, we performed a web-based cross-sectional study.

**Methods:**

The web-based cross-sectional design was applied between June 27 and September 2, 2021. Using convenience sampling, 637 HCWs were recruited from hospitals in Northern Iran (Mazandaran). The HCWs completed self-report questionnaires that included a sociodemographic information form, the 12-item General Health Questionnaire, Impact of the Event Scale-Revised, Risk Perception Questionnaire, and Anxiety Stress Scale‐21. The data were analyzed via descriptive and inferential statistics and univariate/multivariate logistic regression to assess the risk factors linked to each psychosocial consequence.

**Results:**

The results reveal that the COVID-19 pandemic had an adverse psychosocial influence on HCWs, which was already apparent 1.5 years after the crisis began. Based on the results, 71.6%, 55.6%, and 32.3% of HCWs reported having anxiety, depression, and stress symptoms, respectively, since the outbreak of this disease. The logistic regression models displayed that marital status, having children, and working hours with patients were all risk factors of psychosocial impairment.

**Conclusions:**

The outbreak of COVID-19 can be considered an important experience of a bio-disaster resulting in a significant rate of psychiatric problems in HCWs. There is a need for designing and promoting supportive programs to help HCWs cope and to improve their psychosocial state, and the present study has detected for whom psychosocial support may be effective and practical 1.5 years after the primary outbreak. Moreover, detecting and managing concerns and reducing infection-related embarrassment/stigma are essential for improving HCWs’ mental health.

## Introduction

Healthcare workers (HCWs) are essential resources in any country, and their health and wellbeing are key not only for offering constant and useful care facilities to clients, but also for maintaining the health and safety of the workforce and patients [1]. With the outbreak of the coronavirus disease 2019 (COVID-19) pandemic, thousands of HCWs around the world faced tremendous pressure directly or indirectly in caring for patients with the disease. They were met with an increased workload, variation in responsibilities, and intense work-related stress. Meanwhile, they were often limited by inadequate equipment and tools. Despite the fatigue caused by the heavy workload, they confronted the large number of deaths and extensive suffering [2–4]. While HCWs were liable for patient care during the outbreak of COVID‐19, they were also dealing with many health worries, such as the lack of personal protective equipment (PPE), distress of contracting the virus themselves, and loss of work due to illness [8–10]. Based on studies around the world, these risk factors have the potential to enhance the risk of psychological difficulties by 39–71% among HCWs, including depression, anxiety, sleeplessness, and sorrow, and even post-traumatic stress disorder (PTSD) [1, 5–18].

A study in the United States showed elevated rates of depression (48%) and anxiety (33%) among HCWs during the COVID-19 pandemic [8]. A systematic review in Latin American countries found that several studies reported higher scores on anxiety as well as increased depression among HCWs [19]. According to a Canadian meta-analysis, the prevalence of insomnia among HCWs was more than twice that of the general population [20]. Moreover, the results of a study in Ethiopia revealed a 78.3% prevalence of psychological distress and 50.2% prevalence of insomnia among HCWs [21].

Studies in Germany reported levels of anxiety and depressiveness symptoms, increased fear of infecting relatives, and a high rate of PTSD signs among HCWs [22, 23]. A study in Italy indicated that individuals working with COVID-19 patients had extra difficulties with depression, anxiety, and stress. The study reported that 49.38% of HCWs declared PTSD symptoms, 19.80% anxiety, 21.90% high perceived stress, 24.73% severe depression, and 8.27% insomnia [24, 25]. Several studies in Spain also reported high rates of psychological distress and PTSD symptoms among HCWs during the COVID-19 crisis [26–28].

Various studies in Asia have also shown high levels of mental disorders and PTSD symptoms among HCWs due to the COVID-19 pandemic. According to an Asian systematic review, the COVID-19 pandemic caused signs of anxiety, stress, and depression, and the COVID-19 crisis can be regarded as an independent stress element among HCWs [29]. In a study in China, 50% of HCWs reported that they were depressed, 45% reported significant anxiety, and 34% reported experiencing insomnia during the crisis [18]. In other study, in China, the prevalence of depression was 44%, and anxiety 46%. Based on these studies, the frontline HCWs had elevated risks of anxiety and psychological problems compared to those on the sidelines [15, 30]. A study in Pakistan reported that the unexpected role reversal from HCW to patient could lead to frustration, fear of discrimination, anxiety, adjustment issues, helplessness, and stigma among HCWs [31]. A recent study from Qatar reported high perceived anxiety, stress, and PTSD symptoms among HCWs working in intensive care units caring for patients infected with COVID-19, with 71.4% of physicians and 74.4% of nurses reporting that they experienced moderate to severe perceived stress [32]. Based on a survey of Turkish physicians during the period of the first wave of the COVID-19 crisis, anxiety and depression were prevalent in women and related to little work history, long working hours, and little support from coworkers and directors [33].

Different investigations in Iran have also indicated the COVID-19 pandemic’s influence on mental health and shown relationships between the pandemic and several medical issues. According to the findings of several studies in the Iranian context, 42.3% of hospital staff reported having a medium to high rate of anxiety, and 39.6–65.6% displayed medium to high depressive signs [34–40]. For instance, in Fateminia et al.’s study, 39.2% of nurses experienced severe PTSD. Azizi et al. reported that HCWs experienced average rates of physical (47.9%) and psychological anxiety (70.5%) [37], and Hosseini et al. found that 68.42% of Iranian HCWs reported mild to severe PTSD [40].

Stress and the resulting adverse mental health consequences for medical staff affect the quality of care and patient satisfaction [41–44]. Having good mental health is very important for HCWs during a pandemic outbreak in a low-income country like Iran, because the healthcare sector has insufficient human resources. It should be noted that the effective provision of healthcare facilities is dramatically affected by human resources. The COVID-19 pandemic forced all healthcare systems in the world, including in Iran, to pay special consideration to the prerequisites and requirements regarding preparation, delivery, implementation, compensation systems, and other related aspects of human resource management [45–48].

With the aim of ensuring the enhanced management of the COVID-19 crisis in Iran, 41 referral hospitals, 168 hospitals, and healthcare centers with the ability to provide emergency services were devoted to providing COVID-19-related facilities [49]. The Primary Health Care Network (PHC), Iran’s major healthcare system, offers access to primary care in the most remote regions, and more than 10 000 urban healthcare centers (5000) and rural comprehensive healthcare centers (5000) were allocated to track and follow up on suspected and positive cases. Furthermore, about 32 000 volunteer groups were formed within communities and by non-governmental organizations (NGOs) to support the healthcare system and government officials in responding to the COVID-19 crisis [49–51]. In Iran, more than 50 labs, most of them public labs, were mobilized to provide diagnostic facilities with a testing capacity of more than 7000 cases/day, [51]. This happened in a situation where Iran’s infrastructure was already impaired due to the effects of long politico-economic sanctions, and the limited human, physical, and financial resources were noticeable [47, 52, 53].

The important point is that the COVID-19 crisis caused numerous different challenges for Iran’s healthcare system [54]. Due to the sudden outbreak of the epidemic in Iran, policymakers and hospitals did not have sufficient time to adjust to the sudden changes and adapt their response, which caused turmoil in the healthcare system [55]. HCWs appeared to be more susceptible to contracting the disease due to frequent exposure as well as to patients with high viral loads in these encounters. They faced further challenges due to the high workload and pressure, such as physical exhaustion, uncertainty, the psychological load of the disease, conflicting feelings, occupational burnout, challenging relationships, psychological violence, equipment deficiency (e.g., PPE), time limitations, low control, and lack of evidence [56–62]. Consequently, there was an unmet need to tackle the psychosocial disease afflicting HCWs during the challenging situation of the COVID-19 pandemic.

Both patients and HCWs need psychological support in clinical treatment. Thus, given the long duration of the COVID-19 pandemic, the increase in HCWs’ workloads, and the adverse impact on their mental health, the current research was conducted with the aim of studying Iranian HCWs’ psychosocial state and risk perception during the COVID-19 crisis. To this end, Iranian HCWs’ psychosocial condition (anxiety, depression, stress, and PTSD) and risk perception and the risk factors of both during the COVID-19 pandemic were estimated and recorded. The results should also highlight gaps for relevant authorities in addressing the wellbeing, mental health, and psychosocial situation of HCWs during the COVID-19 pandemic. Furthermore, we aimed to identify demographic variables related to mental illness and PTSD among Iranian HCWs during the COVID-19 pandemic.

The findings of this study offer evidence that due to the tense COVID-19 period, HCWs must receive support and suitable psychological interventions in structuring useful therapeutic patterns, including resilience approaches at the individual and organizational levels, to decrease and prevent the psychological consequences of the COVID-19 crisis, particularly PTSD. Planning psychological resilience programs for HCWs (especially those working on the front line) was one of the main priorities during the COVID-19 crisis. Moreover, the findings of this study can be helpful for management units and politicians addressing mental health in their design and implementation of educational courses and seminars to train mental HCWs, such as training courses on stress management, anxiety, and resilience. Lastly, it is essential to launch early, targeted mental health intervention programs across Iran, especially for HCWs. The results of this study are expected to clarify the psychosocial effects of exposure to the recent pandemic as well as the preparation for possible crises caused by the outbreak of other infectious diseases in the future.

## Methods

### Study design and population

A web-based cross-sectional study was conducted on a sample of 637 HCWs chosen via convenience sampling, from June 27 to September 2, 2021, in all cities of Northern Iran, during the fifth and most severe wave of the COVID‐19 crisis. The convenience sample of HCWs included general practitioners (GPs), midwives, clinical staff, nurses, radiology technicians, laboratory technicians, service and cleaning staff, drivers, supervisors, and support team members.

### Inclusion and exclusion criteria

The inclusion criteria included Iranian HCWs directly or indirectly in contact with infected patients in outpatient/inpatient care units, pre-hospital emergency medical centers, and healthcare clinics, and expressing preparedness to contribute to the current study.

The exclusion criteria were HCWs who declared that they had trouble with their internet connection while accessing the questionnaire, those whose questionnaire was incomplete (i.e., they did not answer all the questions), those who did not satisfy the study inclusion criteria, those who could not complete the questionnaire on their phone, or those who were dissatisfied with the number of questions. In addition, we regarded diagnosed physical or mental disorders and a record of psychiatric care as exclusion criteria.

Individuals who were not HCWs were excluded from the current study. Participants gave their explicit consent by filling out an informed consent box before completing the questionnaire. HCWs were initially classified as a direct contact group if their occupation suggested direct contact with people infected with COVID-19. They were classified as an indirect contact group in case of exposure to patient-related cases, such as laboratory samples and equipment [63]. No financial support was given to the HCWs to participate in this study.

### Procedure

First, the purpose of the study was explained to the HCWs invited to participate in the study. If they were willing to participate in the study, starting on February 23, 2022, self-administered online questionnaires were sent to HCWs via WhatsApp, Telegram, and email. If they did not complete the questionnaire within a week, reminders were sent to them to maximize the response rate. This reminder was sent up to two times, 1 week apart. Anyone who did not complete the questionnaire after receiving two reminders was excluded from the study. Questionnaires were collected until April 28, 2022, the estimated peak of the fifth wave of the COVID-19 crisis in Iran.

### Instruments and data collection

The Farsi version of the questionnaire was create on an online platform (https://porsline.ir/online-questionnaire/). The researchers checked the accuracy of the questionnaire and then distributed it online among groups of HCWs working in various therapeutic centers during the COVID‐19 pandemic via common messaging applications, such as Telegram, Short Message Service, WhatsApp, and Instagram. The time required for HCWs to complete the questionnaire was considered 20–25 min. The questionnaire was disseminated to various HCWs groups, and they sent it to other virtual groups of HCWs.

The first page of the web-based survey included a statement explaining the study title and purpose of collecting the HCWs’ data and characteristics. It also briefly explained the questionnaire’s contents and approaches for answering the items. Only completely filled-in questionnaires were considered during the analysis stage, in accordance with the planned instructions until the last stage. The responsible author of the article (MK) regularly checked the number of completed questionnaires by logging into the platform using her username and password. The participants completed five main instruments: the sociodemographic characteristics information form, 12-item General Health Questionnaire (GHQ-12), Impact of the Event Scale-Revised (IES-R), Risk Perception Questionnaire (RPQ), and Anxiety Stress Scale‐21 (DASS‐21).

#### Sociodemographic characteristics information form

The sociodemographic characteristics assessed comprised age (≤ 35; 36–50; > 50), sex (male, female), marital status (single, married, divorced/widowed), having children (yes, no), educational level (under diploma, bachelor, master’s, PhD; general or specialist medical degree/pharmacy), working in isolation units (yes, no), occupation (general or special professional doctor, pharmacist, nurse, midwife, technician, support staff), working hours with COVID patients (0; 1–2; 3–6; > 6 h), type of patient contact (direct; indirect), years of experience (< 5; 5–10; > 10 years), experience of psychiatric disorders (yes; no), and experience of physical illness (yes; no), along with some questions about the COVID‐19 crisis.

#### General Health Questionnaire (GHQ-12)

We applied GHQ-12, established by Goldberg, to measure psychological status [64]. The scale is multidimensional and has items measuring non-psychotic disorders, such as anxiety, social inefficiency, and depression. GHQ-12 was extensively applied to measure mental status during the severe acute respiratory syndrome (SARS) pandemic and, more recently, the COVID-19 pandemic and has been deemed reliable [12, 65–68]. The scale consists of 12 items—six items with negative phrases and six with positive phrases—and scores range from 0 to 36 [69]. Concisely, we implemented the 4-point Likert scale comprising 0 = “Not at all,” 1 = “No more than usual,” 2 = “Rather more than usual,” and 3 = “Much more than usual”. Items worded positively were scored as follows: 0 = “More so than usual,” 1 = “Same as usual,” 2 = “Less so than usual,” and 3 = “Much less than usual”. All items were collected to obtain a total score ranging from 0 to 36 (with a larger score representing a worse state of mental health). We categorized participants with scores above the cut-off point of 12 as having experienced mental distress, as defined earlier [70]. In a previous Iranian study, the reliability of GHQ-12 was demonstrated, and the scores for social inefficiency, mental distress, and general scores were 0.80, 0.78, and 0.82, respectively. The test–retest correlation coefficients among the two subscales of GHQ-12 along with the total scores were from 0.84 to 0.93 [71]. GHQ-12 has been used in various studies in Iran, and its reliability and validity have been confirmed in the Iranian population [69, 72, 73].

#### Depression Anxiety Stress Scale‐21

DASS‐21 is a short version of DASS‐42 [74]. Lovibond et al. used DASS‐21 to determine how common depression, anxiety, and stress are among adults in different places (clinical or nonclinical settings) [75]. The self-report scale of DASS‐21 asks respondents to rate how often and how severe their negative feelings were during the past week on a 4-point Likert scale ranging from 0 = “Did not apply to me at all” to 3 = “Applied to me very much or most of the time”. Generally, in any dimension, the score can have a domain from 0 to 63, and the higher the score, the greater the intensities of stress, depression, and anxiety [76, 77]. Similarly, for the depression dimension, scores from 0 to 9 indicate normal mental health (no signs of depression); scores from 10 to 13 represent mild signs of depression; scores from 14 to 20 represent moderate signs of depression; scores from 21 to 27 reflect severe signs of depression; and scores above 27 indicate extremely severe signs of depression. Regarding the anxiety dimension, scores from 0 to 7 are classified as normal mental health (no anxiety); scores from 8 to 9 demonstrate mild anxiety; scores from 10 to 14 reflect moderate anxiety; scores from 15 to 19 indicate high anxiety; scores from 20 to 27 suggest severe anxiety; and scores above 27 indicate extremely severe anxiety. Furthermore, for the stress dimension, scores from 0 to 14 are normal (without stress); scores from 15 to 18 display slight stress; scores from 19 to 25 demonstrate moderate stress; scores from 2 to 33 exhibit severe stress; and scores above 33 indicate very severe stress [78]. In various investigations, the reliability of DASS‐21 in the dimensions of depression, anxiety, and stress have been confirmed with Cronbach’s alpha values of 0.82, 0.90, and 0.93, respectively [79]. In Iranian research, the reliability of DASS-21 was found to be 0.86, 0.76, and 0.79 for the three dimensions of depression, anxiety, and stress, respectively [80]. DASS-21 has been used in various studies in Iran, and its reliability and validity have been confirmed in the Iranian population [76, 77, 81, 82].

#### Impact of the Event Scale-Revised (IES-R)

Impact of Event Scale-Revised (IES-R) is a 22-item self-report scale that measures subjective distress affected by traumatic events. The 15-item IES is the original instrument but lacks overexcitement signs. IES-R comprises seven extra items concerning the overexcitement signs of PTSD lacking in the initial version of IES. The questions directly match 14 of the 17 DSM-IV signs of PTSD [83]. Participants are asked to consider a specific stressful life event and how much they were bothered or distressed by each difficulty relating to that event during the past 7 days [84]. The scale has demonstrated suitable assessment invariance as a one-factor scale with both Latino and non-Latino peoples [85, 86]. The items are ranked on a 5-point scale ranging from 0 (“Not at all”) to 4 (“Extremely”), with a possible total score ranging from 0 to 88, and the cut-off of 33 demonstrates a high risk of PTSD. Besides, IES-R gives a total score (ranging from 0 to 88), and dimension scores can be assessed for the three subscales of Intrusion, Avoidance, and Overexcitement [87]. IES-R has been used in various studies in Iran, and its reliability and validity have been confirmed in the Iranian population [88–91].

#### Risk perception questionnaire (RPQ)

To measure participants’ risk perception (RP), we developed a self-administered, structured scale on the basis of past studies on epidemics of infectious respiratory diseases [12, 63, 92, 93]. The RP of COVID-19 was measured by 24 concern statements regarding stigma, fear of spreading COVID-19, fear of contracting COVID-19, and workplace-related situations. Each statement had four answer choices ranging from 0 = “Strongly agree” to 3 = “Strongly disagree”. Concern phrases had negative wordings (e.g., “There is no sufficient PPE at my workplace”). A higher score indicates a higher RP. The reliability of RPQ has been confirmed with Cronbach’s alpha values of 0.82 in this study.

### Ethical considerations

This study was accepted by the Ethics Committee of Mazandaran University of Medical Sciences (IR.MAZUMS.REC.1399.002), Sari, Iran. The research objectives were explained to participants, who then signed a web-based informed consent form, and assurance was given to them about the privacy of their data. Questionnaires were only sent to HCWs who were willing to participate in the study and expressed their consent to do so. Moreover, all participants were assured that the collected information was only for use in this study, the researchers would keep the data confidential, and there would be no need to record any identifying information about them.

### Data management and statistical analysis

Data analysis was undertaken using SPSS Statistics software (ver. 24). Furthermore, missing data were assessed and deleted before the analysis. Descriptive analyses were performed to test the participants’ characteristics. Then bivariate analysis was carried out to classify elements regarding high levels of PTSD signs.

Categorical data were summarized by frequencies and percentages, and continuous data were offered as *M* ± SD. In DASS-21, the three dimensions of stress, depression, and anxiety were identified, and each domain was categorized into five classifications: normal, mild, moderate, severe, and very Severe. The scores of GHQ-12 were divided into two categories: high and low. PRQ was divided into three categories: low risk, moderate risk, and high risk. The Chi-square (*χ*^2^) test was applied for comparing between‐group differences. Univariate/multivariate ordinal logistic regression models were applied to uncover the possible predictors of anxiety during the COVID-19 pandemic. Logistic regression was used to analyze the PTSD questionnaire. To analyze the risk perception and DASS-21, first, univariate logistic regression was performed for all variables, and then the variables whose *p*-value was less than 0.2 were entered into the multivariate logistic regression model.

## Results

### Descriptive and bivariate analyses of healthcare workers during the COVID‐19 pandemic

The characteristics of the total sample are displayed in the first column of Table 1. As shown in Table 1, among the 637 HCWs, 473 (74.3%) were female. The mean age was 41.05 ± 0.46 years (range 24–67 years); 277 (43.5%) were aged 36–50 years; and 136 (21.4%) were aged 50 years or older. There were 449 (70.5%) persons who expressed having experienced direct contact with COVID-19 patients; 92 (14.4%) of them worked for more than eight hours with COVID‐19 patients and 73 (about 12%) were working in isolation units. Moreover, 22 (3.5%) had a history of psychiatric disorders.

**Table 1:** Bivariate associations between level of PTS symptoms and related factors (*n* = 637)

High level of PTS symptoms^a^					
Variable	Total:* n* = 637 (% and CI)	No: *n* = 52 (% and CI)	Yes: *n* = 585 (% and CI)	OR (95% CI)	*P*-value
Age, years					
≤ 35	224 (35.2 [31.4, 39.0])	19	205	0.95 (0.44, 2.06)	0.90
36–50	277 (43.5 [39.6 47.4])	22	255	1.02 (0.48, 2.17)	0.96
> 50	136 (21.4 [18.2, 24.7])	11	125	1	
Sex					
Male	164 (25.7 [22.4, 29.3])	12	152	1.17 (0.60, 2.29)	0.65
Female	473 (74.3 [70.7, 77.6])	40	433	1	
Marital status					
Single	189 (29.7 [26.1, 33.4])	16	173	1.01 (0.41, 2.44)	0.99
Married	354 (55.6 [51.6, 59.5])	28	326	1.08 (0.48, 2.46)	0.85
Divorced or widowed	94 (14.8 [12.1, 17.7])	8	86	1	
Having children					
Yes	354 (55.6 [51.6, 59.5])	32	322	0.77 (0.43, 1.37)	0.37
No	283 (44.4 [40.5, 48.4])	20	263	1	
Level of education					
Under diploma	37 (5.8 [5.4, 6.2])	3	34	1.22 (0.32, 4.64)	0.77
Bachelor’s degree	411 (64.5 [62.7, 66.3])	28	383	1.48 (0.71, 3.06)	0.30
Master’s/PhD degree	76 (11.9 [11.1, 12.7])	10	66	0.71 (0.29, 1.77)	0.46
Doctorate (general and specialist doctor)/pharmacy	113 (17.7 [16.6, 18.9])	11	102	1	
Working in isolated units					
Yes	73 (11.5 [9.1, 14.2])	7	66	0.82 (0.35, 1.89)	0.64
No	564 (88.5 [85.8, 90.9])	45	519	1	
Occupation					
General or special professional doctor/pharmacist	114 (17.9 [16.8, 19.0])	12	102	0.5 (0.11, 2.35)	0.38
Nurse/midwife	314 (49.3 [47.4, 51.2])	25	289	0.68 (0.15, 3)	0.61
Technician^b^	173 (27.2 [25.6, 28.7])	13	160	0.72 (0.16, 3.36)	0.68
Support staffs^c^	36 (5.7 [5.2, 6.1])	2	34	1	
Working hours with COVID‐19 patients					
0	191 (30 [26.4, 33.7])	17	174	0.84 (0.34, 2.11)	0.72
1–2	153 (24 [20.7, 27.5])	8	145	1.49 (0.52, 4.26)	0.45
2–4	79 (12.4 [9.9, 15.2])	11	68	0.51 (0.19, 1.38)	0.19
4–6	81 (12.7 [10.2, 15.6])	7	74	0.87 (0.29, 2.60)	0.80
6–8	41 (6.4 [4.7, 8.6])	2	39	1.61 (0.31, 8.09)	0.57
> 8	92 (14.4 [11.8, 17.4])	7	85	1	
Category by patient contact					
Direct contact group	449 (70.5 [66.8, 74.0])	35	414	1.18 (0.64, 2.16)	0.60
Indirect contact group	188 (29.5 [26.0, 33.2])	17	171	1	
Years of experience					
< 5	123 (19.3 [16.3, 22.6])	13	110	0.69 (0.35, 1.37)	0.29
5–10	116 (18.2 [15.3, 21.4])	9	107	0.97 (0.45, 2.11)	0.94
> 10	398 (62.5 [58.6, 66.3])	30	368	1	
History of physical illness					
Yes	60 (9.4 [7.3, 11.9])	4	56	1.27 (0.44, 3.65)	0.66
No	577 (90.6 [88.0, 92.7])	48	529	1	
History of psychiatric disorders					
Yes	22 (3.5 [2.2, 5.2])	2	20	1.13 (0.26, 4.97)	0.87
No	615 (96.5 [94.8, 97.8])	50	565	1	

^a^Percentages, within the low and high PTS symptom level groups, falling into each category of each factor variable, are reported here

^b^Technician: radiology technician, laboratory technician, and operating room technician

^c^Support staffs: cleaners, ambulance drivers, and administrators

About 585 (92% CI [0.89, 0.94]) of the HCWs had high scores for signs of post-traumatic stress (PTS) (IES-R score ≥ 20) in the period of 1.5 years due to their exposure to the COVID-19 crisis (from the beginning of the pandemic to the fifth wave in Iran). About 52 (8% CI [0.06, 0.11]) got a score lower than or equal to 20, showing a low level of PTS. Bivariate analysis was used to investigate the relationship between demographic variables and PTS level (high and low), and none of the studied variables were significantly related to PTS level (*p* > 0.05). On the subject of working in isolation units, history of psychiatric disorders, hours of working with COVID‐19 patients, contact with patients, and history of physical illness were not associated with high levels of PTSD symptoms (Table 1).

### Level of perceived risk of COVID-19 in healthcare workers during the COVID‐19 pandemic

The participants’ greatest worries were about coming into contact with or getting infected with COVID-19, which they discovered in feeling compelled to take care of COVID-19 patients (23.5% CI [20.3, 27.0]), feeling that there was no adequate PPE (23.3% CI [20.0, 26.7]), feeling anxious at work (23.2% CI [20.0, 26.7]), and feeling that they would possibly transmit COVID-19 to family members (22.2% CI [19.0, 25.6]) (Table 2). Some also reported the new COVID-19 regulations (22.9% CI [19.7, 26.4]) and insufficient staffing (22.5% CI [19.3, 25.9]) as challenges and problems. Participants were least worried about feeling they must refrain from going to the workplace to avoid getting infected with COVID-19 (80.3% CI [76.9, 83.2]), not having adequate training in infection prevention and control (IPC) (79.8% CI [76.4, 82.8]), and lack of family care if they contracted COVID-19 (78.8% CI [75.4, 81.9]). More than a fifth of HCWs stated that they would feel embarrassed to tell their family if they got infected with COVID-19 (22% CI [18.8, 25.4]), were concerned that the workplace did not have a clear and documented plan to respond to the outbreak (21.4% CI [18.2, 24.7]), and felt that they had to change their work because of the threat of getting infected with COVID-19 (20.9% CI [17.8, 24.2]) (Table 2).

**Table 2:** Concerns of HCWs regarding the COVID-19 outbreak during the early phase of the epidemic, Northern Iran (*N* = 637)

Concern statements	Responses to concern statements (*N* = 637)			
	Number		Percent	
	Low concern	High concern	Low concern (CI)	High concern (CI)
Fear of contracting COVID-19 at workplace				
I would feel endangered if a colleague got infected with COVID-19	490	147	76.9 (73.5, 80.1)	23.1 (19.9, 26.5)
I am at risk of getting infected with COVID-19	500	137	78.5 (75.1, 81.6)	21.5 (18.4, 24.9)
I feel anxious at work	489	148	76.7 (73.3, 80.0)	23.2 (20.0, 26.7)
I am unsafe at work	501	136	78.7 (75.3, 81.8)	21.4 (18.2, 24.7)
I will eventually get infected with COVID-19	491	146	77.1 (73.6, 80.3)	22.9 (19.7, 26.4)
Being absent will reduce my chances of getting infected with COVID-19	492	145	77.2 (73.8, 80.4)	22.7 (19.6, 26.2)
I feel helpless about getting infected with COVID-19	500	137	78.5 (75.1, 81.6)	21.5 (18.4, 24.9)
I feel I should avoid going to work to avoid getting infected with COVID-19	511	126	80.3 (76.9, 83.2)	19.8 (16.8, 23.1)
I do not feel safe even when I use standard IPC measures	498	139	78.2 (74.8, 81.3)	21.9 (18.7, 25.2)
I feel I should change my job because of the threat of getting infected with COVID-19	504	133	79.1 (75.8, 82.2)	20.9 (17.8, 24.2)
Perceived workplace risks and conditions				
My workplace would not support me if I get infected with COVID-19	500	137	78.4 (75.1, 81.6)	21.5 (18.4, 24.9)
The COVID-19 outbreak has increased my workload	502	135	78.8 (75.4, 81.9)	21.2 (18.1, 24.6)
The workload and staffing do not match	494	143	77.5 (74.1, 80.7)	22.5 (19.3, 25.9)
There is insufficient PPE	489	148	76.8 (73.3, 80.0)	23.3 (20.0, 26.7)
I have not received sufficient training in IPC	508	129	79.8 (76.4, 82.8)	20.3 (17.2, 23.6)
I feel overwhelmed by new COVID-19 regulations	491	146	77.1 (73.6, 80.3)	22.9 (19.7, 26.4)
I am not confident about the IPC measures	498	139	78.2 (74.8, 81.3)	21.9 (18.7, 25.2)
There is no clear outbreak response plan	501	136	78.6 (75.3, 81.8)	21.4 (18.2, 24.7)
Fear of spreading COVID-19				
I should social distance more than non-HCWs	498	139	78.2 (74.8, 81.3)	21.8 (18.7, 25.2)
I will likely transmit COVID-19 to family members	496	141	77.9 (74.4, 81.0)	22.2 (19.0, 25.6)
Stigma against self (internal) and others (external)				
I would feel ashamed disclosing to colleagues if I get infected with COVID-19	492	145	77.2 (73.8, 80.4)	22.8 (19.6, 26.2)
Family will not look after me if I get infected with COVID-19	502	135	78.8 (75.4, 81.9)	21.2 (18.1, 24.6)
I feel forced to care for COVID-19 patients	487	150	76.4 (73.0, 79.7)	23.5 (20.3, 27.0)
I would feel embarrassed to tell my family if I get infected with COVID-19	497	140	78 (74.6, 81.2)	22.0 (18.8, 25.4)

*IPC* infection prevention and control, *HCW* healthcare worker, *COVID-19* coronavirus disease, *PPE* personal protective equipment

### Ordinal logistic regression models for predictors of perceived risk in healthcare workers during the COVID‐19 pandemic

The binomial logistic regression method was implemented to test the relationships between demographic variables and different components of the RPQ. First, univariate analysis was used, and the variables whose *p*-value was less than 0.30 were included in the multivariate analysis, and other variables were excluded from the multivariate model. In the univariate analysis, the relationship between working in an isolation unit and the level of risk perception was significant. For the group of HCWs who worked in isolation units, the chance of understanding their risk was 2.31 times that of those who did not work in isolation units. For the multivariate analysis, the variables of marital status, working in an isolation unit, job, working hours, and type of patient contact were entered into the model. In the multivariate analysis, the relationship between working in an isolation unit and the level of risk perception was significant. For HCWs who worked in an isolation unit, the chance of understanding their risk was 2.36 times that of people who did not work in an isolation unit (Table 3).

**Table 3:** Ordinal logistic regression for predictors of perceived risk among participants

Perceived risk				
Variables	Univariate		Multivariate	
	OR (95% CI)	*P*	OR (95% CI)	*P*
Age				
≤ 35	0.86 (0.56, 1.33)	0.51		
36–50	0.96 (0.64, 1.46)	0.86		
> 50	Ref.	–		
Sex				
Male	0.93 (0.64, 1.33)	0.68		
Female	Ref.	–		
Marital status				
Single	1.48 (0.87, 2.53)	0.15	1.54 (0.9, 2.65)	0.12
Married	1.61 (0.98, 2.64)	0.06	1.55 (0.94, 2.56)	0.09
Divorced or widowed	Ref.	–	Ref.	–
Having children				
Yes	1.13 (0.82, 1.55)	0.46		
No	Ref.	–		
Level of education				
Under diploma	1.08 (0.51, 2.32)	0.84		
Bachelor’s degree	1.07 (0.7, 1.65)	0.75		
Master’s/PhD degree	1.56 (0.88, 2.79)	0.13		
Doctorate (general and specialist doctor)/pharmacy	Ref.	–		
Working in isolated units				
Yes	2.3 (1.44, 3.65)	< 0.001	2.36 (1.45, 3.83)	< 0.001
No	Ref	–	Ref	–
Occupation				
General or special professional doctor/pharmacists	0.99 (0.46, 2.14)	0.97	0.82 (0.37, 1.84)	0.64
Nurse/midwife	1.1 (0.54, 2.24)	0.80	0.91 (0.42, 1.95)	0.80
Technician^a^	1.12 (0.53, 2.34)	0.77	1.06 (0.49, 2.3)	0.89
Support staffs^b^	Ref.	–	Ref	–
Working hours with COVID patient				
0	0.83 (0.52, 1.31)	0.42	1.26 (0.19, 8.32)	0.81
1–2	0.99 (0.61, 1.58)	0.95	0.99 (0.57, 1.73)	0.98
2–6	1.28 (0.81, 2.03)	0.29	1.29 (0.8, 2.09)	0.30
> 6	Ref.	–	Ref.	–
Category by patient contact				
Direct	1.32 (0.93, 1.88)	0.12	1.5 (0.24, 9.45)	0.67
Indirect	Ref.	–	Ref.	–
Years of experience				
< 5	0.92 (0.61, 1.39)	0.70		
5–10	0.88 (0.58, 1.35)	0.57		
> 10	Ref.	–		
History of physical illness				
Yes	1.16 (0.68, 1.97)	0.58		
No	Ref.	–		
History of psychiatric disorders				
Yes	0.93 (0.39, 2.24)	0.87		
No	Ref.	–		

^a^Technicians: operating room, laboratory, radiology

^b^Support staffs: cleaners, ambulance drivers, and administrators

### Level of psychological distress in healthcare workers during the COVID‐19 pandemic

The HCWs’ mean GHQ-12 distress score was 11.44 (SD ± 8.14). The scores were between 0 and 36; 421 HCWs (66.1% CI [0.62, 0.69]) scored below the average and 216 of them (33.9% CI [0.31, 0.37]) scored above the average. The most expressed indexes from the GHQ-12 scale with a score of more than 1 were not enjoying daily activities (79% CI [75.4, 81.9]) and not feeling useful in society (79% CI [75.4, 81.9]). After them, the items constantly feeling under stress (78% CI [74.9, 81.5]), worrying about losing sleep due to COVID-19 (78% CI [74.1, 80.7]), not feeling reasonably happy (77% CI [73.0, 79.7]), inability to concentrate on tasks (76% CI [72.5, 79.3]), feeling worthless (76% CI [72.3, 79.1]), lost confidence (75% CI [71.3, 78.2]), feeling unhappy and depressed (75% CI [71.2, 78.1]), inability to overcome difficulties (75% CI [71.2, 78.1]), inability to face up to problems (75% CI [71.2, 78.1]), and inability to make decisions (74% CI [71.7, 78.5]) (Table 4).

**Table 4:** Frequency of GHQ-12 items among healthcare workers in tertiary referral hospitals during the early phase of the epidemic, Iran, (*N* = 637)

Items^b^ from GHQ-12 questionnaire^c^	Percentage of score responses (*N* = 637)				
	0	1	2	3	2 or 3^a^
Not enjoying daily activities	234	268	92	43	135
Feeling constantly under stress	210	289	96	42	138
Not feeling reasonably happy	229	258	116	34	150
Feeling unhappy and depressed	227	249	129	32	161
Cannot overcome difficulties	197	279	121	40	161
Cannot concentrate on tasks	215	269	112	41	153
Worrying about losing sleep due to COVID-19	204	290	102	41	143
Cannot face up to problems	202	274	122	39	161
Lost confidence	219	258	124	36	160
Feeling worthless	231	252	117	37	154
Not capable of making decisions	224	255	120	38	158
Not feeling useful in society	225	277	104	31	135

*GHQ* General Health Questionnaire

^a^A higher score signifies psychologically distressed state

^b^All items were asked about for the period of the past 1 month

^c^GHQ-12 items as proposed by Goldberg [19]

### Prevalence of depression, anxiety, and stress pursuant to DASS‐21 in healthcare workers during the COVID‐19 pandemic

The findings from DASS‐21 are detailed in Table 5. These findings show that 55.6% of the staff reported levels of depression during the COVID-19 crisis, and the majority of them expressed having mild depression (18.5%). The prevalence of anxiety in the participants was appraised at 71.6%, and among them, the majority stated that they had extremely intense anxiety (26.4%). Based on Table 4, about 32.3% of HCWs reported experiencing levels of stress during the COVID-19 pandemic and between them, most of the sample reported suffering moderate stress (13.5%) (Table 5).

**Table 5:** Prevalence of depression, anxiety, and stress based on the DASS‐21

Questionnaire domains and severity					
DASS	Severity of depression, anxiety, and stress, *N* = 637 (%)				
	Normal	Mild	Moderate	Severe	Very severe
Depression	283 (44.4%)	118 (18.5%)	94 (14.8%)	75 (11.8%)	67 (10.5%)
Anxiety	181 (28.4%)	101 (15.9%)	136 (21.4%)	51 (8%)	168 (26.4%)
Stress	431 (67.7%)	41 (6.4%)	86 (13.5%)	46 (7.2%)	33 (5.2%)

### Ordinal logistic regression models for predictors of depression, anxiety, and stress in healthcare workers

Table 6 displays the predictors of depression, anxiety, and stress in participants. Ordinal logistic regression was implemented to test the relationship between demographic variables and different components of DASS-21. First, univariate analysis was used, and the variables whose *p*-value was less than 0.30 were included in the multivariate analysis, while other variables were excluded from the multivariate model (Table 6).

**Table 6:** Ordinal logistic regression for predictors of DASS-21 among participants

Variables	Depression				Anxiety				Stress			
	Univariate		Multivariate		Univariate		Multivariate		Univariate		Multivariate	
	OR (95% CI)	*P*	OR (95% CI)	*P*	OR (95% CI)	*P*	OR (95% CI)	*P*	OR (95% CI)	*P*	OR (95% CI)	*P*
Age												
≤ 35	0.8 (0.55, 1.18)	0.27			0.9 (0.61, 1.31)	0.57			0.77 (0.5, 1.19)	0.24		
36–50	0.8 (0.55, 1.16)	0.23			0.85 (0.59, 1.23)	0.39			0.76 (0.5, 1.15)	0.20		
> 50	Ref.	–	Ref.	–	Ref.	–	Ref.	–	Ref.	–	Ref.	–
Sex												
Male	0.91 (0.65, 1.25)	0.55			0.98 (0.71, 1.35)	0.90			0.89 (0.61, 1.3)	0.56		
Female	Ref.	–	Ref.	–	Ref.	–	Ref.	–	Ref.	–	Ref.	–
Marital status												
Single	1.23 (0.77, 1.95)	0.38	1.74 (1.01, 2.99)	0.046	1.04 (0.67, 1.61)	0.87			1.25 (0.73, 2.13)	0.42		
Married	1.52 (1, 2.33)	0.051	1.33 (0.86, 2.06)	0.20	1.04 (0.69, 1.56)	0.85			1.36 (0.83, 2.24)	0.22		
Divorced/widowed	Ref.	–	Ref.	–	Ref.	–	Ref.	–	Ref.	–	Ref.	–
Having children												
Yes	1.49 (1.12, 1.99)	0.006	1.8 (1.16, 2.8)	0.009	1.25 (0.94, 1.65)	0.12	1.23 (0.92, 1.63)	0.16	1.39 (1, 1.93)	0.052	1.42 (1.01, 2)	0.04
No	Ref.	–	Ref.	–	Ref.	–	Ref.	–	Ref.	–	Ref.	–
Level of education												
Under diploma	0.92 (0.47, 1.79)	0.80	17.4 (16.72, 18.08)	< 0.001	0.6 (0.31, 1.16)	0.13	16.21 (15.53, 16.91)	< 0.001	0.66 (0.31, 1.43)	0.29	8.93 (0.25, 318.88)	0.23
Bachelor’s degree	0.62 (0.43, 0.91)	0.01	17.01 (16.58, 17.45)	< 0.001	0.46 (0.32, 0.67)	< 0.001	15.94 (15.51, 16.37)	< 0.001	0.54 (0.36, 0.82)	0.004	7.57 (0.2, 292.05)	0.28
Master’s/PhD degree	1.17(0.7, 1.97)	0.55	17.59 (16.98, 18.2)	< 0.001	0.73 (0.43, 1.23)	0.23	16.35 (15.75, 16.96)	< 0.001	1.1 (0.62, 1.92)	0.75	14.29 (0.36, 568.58)	0.16
Doctorate (general and specialist doctor)/pharmacy	Ref.	–	Ref.	–	Ref.	–	Ref.	–	Ref.	–	Ref.	–
Working in isolated units												
Yes	1.15 (0.74, 1.8)	0.51			1.13 (0.73, 1.75)	0.57			1.23 (0.75, 2.01)	0.42		
No	Ref.	–	Ref.	–	Ref.	–	Ref.	–	Ref.	–	Ref.	–
Occupation												
General or special professional doctor/pharmacist	1.2 (0.61, 2.36)	0.59	17.54 16.75, 18.8)	< 0.001	1.81 (0.93, 3.54)	0.08	16.8 (16.5, 17.12)	< 0.001	1.71 (0.78, 3.76)	0.18	15.63 (0.42, 582.9)	0.14
Nurse/midwife	0.87 (0.46, 1.62)	0.65	1.14 (0.8, 1.62)	0.48	0.95 (0.52, 1.76)	0.88	1.3 (0.89, 1.91)	0.17	1.16 (0.56, 2.43)	0.69	1.39 (0.88, 2.21)	0.16
Technician^a^	0.72 (0.37, 1.38)	0.13	–	–	0.75 (0.39, 1.42)	0.37	–	–	0.79 (0.36, 1.73)	0.56	–	–
Support staffs^b^	Ref.	–	Ref.	–	Ref.	–	Ref.	–	Ref.	–	Ref.	–
Working hours with COVID patient												
0	1.43 (0.95, 2.15)	0.09			1.51 (1.02, 2.25)	0.04	1.42 (0.93, 2.18)	0.11	1.57 (0.97, 2.52)	0.06	2.27 (0.38, 13.67)	0.37
1–2	1.26 (0.82, 1.93)	0.29			1.25 (0.82, 1.89)	0.30	1.23 (0.76, 2)	0.39	1.14 (0.68, 1.89)	0.62	1.24 (0.68, 2.25)	0.48
2–6	1.37 (0.89, 2.09)	0.15			1.42 (0.94, 2.14)	0.10	1.37 (0.89, 2.1)	0.15	1.52 (0.93, 2.48)	0.10	1.55 (0.92, 2.59)	0.10
> 6	Ref.	–	Ref.	–	Ref.	–	Ref.	–	Ref.	–	Ref.	–
Category by patient contact												
Direct	0.87 (0.64, 1.19)	0.38			0.81 (0.6, 1.1)	0.19			0.8 (0.56, 1.13)	0.20	1.39 (0.25, 7.9)	0.71
Indirect	Ref.	–	Ref.	–	Ref.	–	Ref.	–	Ref.	–	Ref.	–
Years of experience												
< 5	0.93 (0.64, 1.34)				1.03 (0.72, 1.48)	0.85			0.98 (0.64, 1.51)	0.94		
5–10	1.21 (0.83, 1.76)				1.29 (0.89, 1.86)	0.18			1.25 (0.82, 1.91)	0.30		
> 10	Ref.	–	Ref.	–	Ref.	–	Ref.	–	Ref.	–	Ref.	–
History of physical illness												
Yes	1.11 (0.68, 1.79)	0.68			1.22 (0.76, 1.97)	0.40			1 (0.57, 1.74)	0.99		
No	Ref.	–	Ref.	–	Ref.	–	Ref.	–	Ref.	–	Ref.	–
History of psychiatric disorders												
Yes	1.17 (0.54, 2.52)	0.70			1.1 (0.52, 2.35)	0.80			1.28 (0.55, 2.99)	0.57		
No	Ref.	–	Ref.	–	Ref.	–	Ref.	–	Ref.	–	Ref.	–

^a^Technicians: operating room, laboratory, radiology

^b^Support staffs: cleaners, ambulance drivers, and administrators

For depression, the univariate analysis results indicated that the chance of depression in those who had children was 50% higher than in those with no children, and this chance was 40% lower in people with a bachelor’s education than in people with a general or specialist medical degree. For the multivariate analysis, the variables of marital status, having children, education level, and occupation were entered into the model, and the results specified that in the presence of other variables in the model, the chance of depression in single people was 74% higher than in those who were divorced or separated. This chance was 80% higher in those who had children (OR = 1.8, 95% CI [1.16–2.8], *p* = 0.009) than in those with no children. The chance of developing depression among those with under diploma (OR = 17.4, 95% CI [16.72–18.08], *p* < 0.001), a bachelor’s degree: (OR = 17.01, 95% CI [16.58–17.45], *p* < 0.001), and a master’s/PhD (OR = 17.59, 95% CI [16.98–18.2], *p* < 0.001) was higher than in people with a general or specialist medical degree, and based on occupation, doctors and pharmacists (OR = 17.54, 95% CI [16.75–18.8], *p* < 0.001) had a greater chance of developing depression than support staff did (Table 6).

For anxiety, the results of the univariate analysis indicated that the chance of anxiety in people with bachelor’s education was 54% less than people with general and specialist medical education, and the chance of anxiety in those who did not work directly with patients was 51%. This was higher than for those who worked with patients for 6 h. For the multivariate analysis, the variables of having children, education level, occupation, and working hours with patients were entered into the model, and the results showed that in the presence of other variables in the model, the chance of suffering from anxiety was higher in all educational levels (under diploma: OR = 16.21, 95% CI [15.53–16.91], *p* < 0.001; bachelor’s degree: OR = 15.94, 95% CI [15.51–16.37], *p* < 0.001; master’s/PhD: OR = 16.35, 95% CI [15.75–16.96], *p* < 0.001) compared to HCWs with a general or specialist medical degree, but the chances of suffering from anxiety were higher among general or specialist medical doctors and pharmacists (OR = 16.8, 95% CI [16.5–17.12], *p* < 0.001) than support staff (Table 6).

In addition, the univariate analysis results indicated that the chance of having stress in those who had children was 39% higher than in those who did not have children, and this chance was 46% lower in people with a bachelor’s education than in people with a general and specialist medical education. In those who did not work directly with patients, the odds of stress were 57% higher than in those who worked with patients for more than six hours. For the multivariate analysis, the variables of having children, education, job, working hours with patients, and the way of having contact with patients were entered into the model, and the results showed that in the presence of other variables, the chance of depression in those who had children was 42% higher than in those with no children (OR = 1.42, 95% CI [1.01–2], *p* = 0.04) (Table 6).

## Discussion

This study aimed to determine the psychosocial status of Iranian HCWs and their risk perception during the fifth and acute wave of the COVID-19 pandemic. The study also aimed to uncover demographic variables related to mental health, and specifically PTSD, among Iranian HCWs during the COVID-19 pandemic.

Our results suggest that high levels of PTSD symptoms persisted among HCWs 1.5 years after the beginning of the COVID-19 crisis in Iran. According to prior research on survivors of a disaster, more than three-quarters suffer from PTSD for about 1 year after a disaster [94, 95]. Consistent with our results, Green et al.’s study [96], which aimed at identifying demographic, work-related, and other predictors of clinically significant PTSD, depression, and anxiety during the COVID-19 pandemic in UK frontline HSCWs (*n* = 1194), reported high clinical distress in frontline HCWs during the first wave of the COVID-19 outbreak, with over 57% of HCWs meeting the threshold for PTSD, anxiety, and/or depression. Consequently, about a third of them used substances, such as alcohol, hookah, and cigarettes, more than the average to cope with these mental problems [96]. Contrary to these results, Li et al.’s [97] study, aimed at evaluating vicarious traumatization scores via a mobile app-based questionnaire in 234 frontline and 292 non-frontline HCWs aiding in COVID-19 control, reported that frontline HCWs experienced less stress and anxiety than non-frontline HCWs, as frontline HCWs indicated stronger psychological endurance [97].

It is worth considering that when someone’s PTSD signs continue for more than 6 months after an event, they are more likely to remain in that state for a long time [98, 99]. The reason for these contradictory results may be due to different surveys in different waves of the pandemic. The authors argue that this result may be understood by considering that frontline HCWs are voluntarily chosen and provided with enough psychological and mental preparation for handling a crisis. Furthermore, the studied frontline HCWs are generally middle-level, backbone personnel with job skills and mental and psychosocial capacity.

Our results show that the rates of stress, depression, and anxiety in the above-mentioned persons measured via DASS‐21 were medium to high. In different studies, the intensity of anxiety, stress, and depression has been reported differently (from mild to severe). Khanal et al. [17], who aimed to identify factors associated with anxiety, depression, and insomnia among 475 HCWs involved in the COVID-19 response in Nepal, reported that the anxiety rate in hospital personnel was 42% and the rate of depressive signs was 37.5% [17]. Que et al. [15], who aimed at investigating the prevalence of psychological problems and related factors in 2285 Chinese HCWs, reported that 46.04% of workers had symptoms of depression, 46.04% had symptoms of anxiety, 28.75% had psychological problems, and 44.37% had experienced symptoms of insomnia during the crisis [15]. Lai et al. [18], who aimed to assess the magnitude of mental health outcomes and associated factors among 1257 HCWs (in 34 hospitals) treating patients exposed to COVID-19 in China, reported 44.7%, 50.7%, and 36.1% prevalence of self-reported symptoms of anxiety, depression, and insomnia, respectively [18].

Suryavanshi et al. [100], who aimed to assess the mental health and quality of life (QoL) of 197 Indian HCWs, reported that the HCWs’ mental health condition was poor. A large proportion reported symptoms of depression (92, 47%), anxiety (98, 50%), and low QoL (89, 45%) [100]. Furthermore, Şahin et al. [101], who aimed to evaluate the prevalence of depression, anxiety, distress, insomnia, and related factors in 939 Turkish HCWs during the COVID-19 pandemic, found that the prevalence of anxiety, depression, insomnia, and distress was 60.2%, 77.6%, 50.4%, and 76.4%, respectively [101]. The frequency of mental health problems was different in the aforementioned studies. This may be due to the different scales used in the studies, the investigated waves of the disease, or the sample sizes used. Moreover, differences are likely due to the dissimilar cultural characteristics in support facilities between Iran and other countries, like China. However, in some cases, China has reported higher levels of stress and anxiety among HCWs, which could be because the disease was first reported in China. Another reason for this contradiction in the results can be that HCWs working on the front line, compared to others, had direct contact with patients suffering from COVID-19 and committed themselves to delivering the right quality of care for this group of patients with their background of significant exposure to psychiatric problems and other challenges.

Besides, our findings reveal that the rates of depression, stress, and anxiety in the sample were significantly associated with risk factors, such as having children/marital status, working hours with patients, and having children/working hours with patients, respectively. Some other studies have recognized age [100], psychiatric disease history [17, 101], stressful events in the workplace and marital status [100], female gender [100–102], job burnout, increasing workload without considering the individual’s ability, using unfavorable coping methods, and having symptoms of physical diseases (e.g., respiratory or digestive diseases) [103] as risk factors for mental illness among HCWs in different countries. Therefore, there is a need for reflection and planning to solve the aforementioned problems (especially structural problems) that can cause psychosocial problems in HCWs.

In line with the results of our study, other studies have reported mental distress in HCWs, but they have identified different rates (according to the GHQ-12 scale). For example, the mental distress rate in Chinese HCWs was reported to be from moderate to severe (39–71% in different studies) [12, 18]. Dai et al. [12] aimed to investigate the risk perception and immediate psychological state of 4357 Chinese HCWs in the early stage of the COVID-19 epidemic, and they found a 39.1% prevalence of psychological distress especially among those working in Wuhan, participating in frontline treatments, having been isolated, and having infected family members or colleagues [12]. In addition, in a study by Lai et al. [18] with aim of assessing the magnitude of mental health outcomes and associated factors among 1257 Chinse HCWs during COVID-19, the prevalence of psychological distress was reported to be 71.5%. They identified that women nurses, frontline healthcare workers, and those working in Wuhan had more extreme scores for all measurements of psychological distress symptoms than other HCWs [18].

Similarly, Migisha et al. [104], who aimed to assess the risk perception and immediate psychological state of 335 HCWs in referral hospitals involved in the management of COVID-19 patients in Uganda early in the pandemic, detected mental distress in 44% of HCWs. The most common perceived distress included fear of workplace infection (81%), stigma from coworkers (79%), lack of support at work (63%), and low access to PPE (56%) [104]. The difference in the psychological distress rates of our study and others, especially studies conducted in China, may be due to the different times at which the studies were conducted. The current study assessed the rate of mental illness among HCWs during the fifth wave of COVID-19, but other studies assessed it at the beginning of the first wave [105, 106]. The COVID-19 epidemic had significant effects on the mental health of HCWs and caused many emotional and psychological problems in them due to the lack of experience in facing this deadly infection, lack of preparation in facing the psychological problems caused by it, and the lack of sufficient control approaches/strategies to prevent infection.

A significant relationship was found between Iranian HCWs’ perceived risk of getting infected with COVID-19 and psychosocial distress. Moreover, the findings of our study reveal that the HCWs’ level of risk perception was significantly associated with working in an isolation unit. Thus, the HCWs who worked in isolation units had the opportunity to perceive a higher risk than the other HCWs. These results are both predictable and in accordance with various studies on the distress of HCWs in times of epidemics of other respiratory infections [12, 63, 92, 107–109]. Therefore, the psychosocial distress of HCWs may be caused by the way they deal with problems related to personal safety, fear of contaminating others or themselves, and their sense of socio-occupational duty. Worries and fears about the care of HCWs or their relatives, variations in workstation dynamics, and seclusion may be important causes of distress. Our results suggest the necessity of using appropriate and timely interventions to reduce the rates of concern in Iranian HCWs and restore their psychosocial health.

About a quarter of HCWs reported insufficient access to PPE, and about three-quarters of them felt safe once IPC measures and actions were implemented. The rate of mental distress of Iranian HCWs about getting infected with COVID-19 may have been exacerbated during problems caused by a lack of PPE. A lack of protective tools, like PPE, can result in compromised working situations, a sense of insecurity, and increased contact with COVID-19 cases. As a significant number of COVID-19 cases are asymptomatic [110], a lack of a suitable sense of safety among HCWs might raise their psychological distress and disturb their mental health. One of the main challenges in controlling the COVID-19 pandemic was the great lack of PPE [111] in light of the highly communicable disease in already compromised healthcare systems lacking materials and PPE. For example, many countries suffered PPE deficiencies during the Ebola epidemic [112–115]. Ensuring that emergency medical equipment is proper and sufficient for the national system during a pandemic is an essential public health emergency response [111]. HCWs, in the face of the COVID-19 pandemic, were concerned about the risks of infection and lack of PPE, resulting in psychological distress; therefore, more measures need to be taken. In this regard and based on Iran’s conditions for providing PPE, it is appropriate to create an emergency reserve medical materials schedule to guarantee the provision of supplies and PPE based on conditions, variety, need, quantity, and quality. Thus, there is a need for inter-sectoral coordination between the Ministry of Health (MoH) of Iran and other organizations, like industries. This coordination is very important for providing human resource management, PPE, and regular training to maintain and improve the physical and mental health of HCWs.

Another finding of the study concerns the intensified workloads and insufficient employment that caused mental distress in HCWs. George et al. [116] in a study aimed at describing the challenges, experience, and coping of HCWs in delivering healthcare in an urban slum in India during the first 40 days of the COVID-19 outbreak, reported that the majority of the HCWs (75%) experienced fear at some point. The HCWs mentioned distracting themselves with entertainment (20.3%) and spending more time with family (39.1%) as approaches to emotional regulation. They believed that the high workload, tiredness, fear of death, concern about stigma in the slums and possible violence, and the guilt of infecting their valued ones emerged as the main factors of stress among them [116]. Arnetz et al. [117] reported that U.S. HCWs were tired and felt helpless in the hospitals during the early phases of the COVID-19 pandemic, as they did not have faith in the management and as there were insufficient sources of support for them during the pandemic. Lee and Lee [118] stated that Korean HCWs caring for COVID-19 patients had burnout and PTSD symptoms. In other words, they felt tired and upset that they could not cope with their PTSD. This would disrupt their relationships with colleagues and patients and also reduce the quality of the care services they provided [118]. Ardabili et al. [119] reported in their qualitative study that Iranian HCWs had difficulty coping with their emotional concerns resulting from the COVID-19 pandemic and thus experienced burnout. Their results indicated excessive work demands along with a lack of work resources and reduced control over the working conditions. They also noted that the failure in treating patients and the feeling of giving unnecessary care in this situation could lead to moral discomfort among HCWs, which is associated with burnout and the decision to be absent or leave the workplace [119]. Therefore, managers could arrange for HCWs to have shorter working hours, rotating periods for HCWs employed in high-risk sectors, and/or rest via break times, when possible, to boost the HCWs’ self-esteem during a crisis. In situations where reducing working hours during pandemics is not an option, managers could, for instance, offer bonus pay for extra hours worked and provide free meals for HCWs during their shifts. Such measures are mostly critical during pandemics with multiple waves, when HCWs will sense the long-term influences of overwork. Likewise, general management can reduce HCWs’ psychosocial distress [119, 120]. This can help in easing HCWs’ worries. Therefore, it is possible to act directly.

Most of the participants stated that they would feel ashamed if they were obliged to disclose to their coworkers that they were infected with COVID-19. Managers should try to reduce the psychosocial pressure and effects on HCWs if they are infected with COVID-19 and consider this disorder [mental state] as caused by work injury, because it may cause HCWs to worry that they will not receive help and support from their workplace if they contract COVID-19. Incidentally, the MoH (at the top level) or the workplace (at the lower level) can consider implementing a policy that, in addition to providing healthcare and compensation to HCWs, declares that they will not lose their jobs if they contract COVID-19. This is important for contract, corporate, and part-time employees. Moreover, peer support networks and programs can be run to reduce HCWs’ psychological distress. Therefore, it is better for HCWs to be encouraged to use such facilities without worrying about stigma, embarrassment, or losing their jobs. These approaches can decline the psychological effects in the workplace during a crisis [120].

The current results show that a significant proportion of HCWs had insomnia and felt worthless, causing ongoing psychological distress, as reported regarding previous epidemics, such as the SARS outbreak [121]. In Jahormi’s study, aimed at investigating the sleep quality of frontline healthcare workers (FLHCWs) in Bahrain during COVID-19, it was shown that the prevalence of insomnia was higher in HCWs compared to the general population. Specifically, 75% of the FLHCWs reported poor sleep, 85% reported moderate to severe stress, and 61% reported both poor sleep quality and moderate to severe stress [122]. Persistent or constant stress, like during the COVID-19 crisis, may have a more harmful influence on sleep, causing sleep disorders, insomnia, and other difficulties. These findings highlight the profound application of support strategies to control psychological distress among HCWs and increase their resilience during the COVID-19 crisis. Supportive methods that are suitable and necessary for decreasing insomnia include decreasing the times of work shifts and workload, employing new HCWs, using mental health experts to offer advice, and launching counseling sessions with easy access to the counselor. Further research should be done to evaluate the mental health consequences associated with COVID-19 in HCWs to implement appropriate interventions to reduce mental disorders. Meanwhile, Iran’s MoH launched nationwide psychosocial support links, including web-based or phone-based counseling/treatment facilities for HCWs, as part of the response to the feedback it received about the pandemic.

## Limitations

Our study faced important limitations, although it also had many strengths, a few of which are mentioned here. First, we used self-reports of psychosocial state and perceived risk of COVID-19; consequently, the results may be susceptible to count response bias, like social desirability (SDR), which can decline with self-administered scales. This may have resulted in an understated rate of mental health distress in the sample, possibly causing our association to be invalid.

Second, our conclusions are based on cross-sectional data, and the psychosocial status and risk perception of the HCWs participating in the study were not investigated before the outbreak of COVID-19. Therefore, it was not possible to accurately assess whether the results relate to COVID-19 and not routine healthcare job stress, and causal relationships between variables could not be determined. Future studies should collect follow-up data at multiple time points to assess the longitudinal variation in the association between these factors at different stages of the COVID-19 pandemic and after the pandemic. Third, the present study was limited by its cross-sectional design, and no comparative analysis was performed with other HCWs from hospitals that did not care for COVID-19 patients. Thus, we cannot prove any causal relationship between the perceived risk of COVID-19 and signs of PTS. Thus, we were not able to compare the psychosocial distress levels among groups.

Fourth, only those HCWs who had access to the Internet and social media were included in the study, which may not represent a comprehensive sample of all HCWs working in hospitals. Fifth, the use of the modified Farsi version available of IES-R to assess PTS was a limitation. Although IES-R assesses levels of PTS signs, it is not self-diagnostic for PTSD. Researchers should be careful when comparing the findings of the present study with subsequent studies using IES-R or other questionnaires for assessing signs of PTS.

Sixth, considering that the study was conducted during the fifth wave of COVID-19 in Iran, another limitation was the difficulty in accessing all HCWs and using other sampling methods (e.g., stratified or systematic random sampling). Therefore, we employed a convenience sampling method. While convenience sampling can be practical, it may introduce bias and limit the generalizability of the findings. Thus, the repeatability of this study must be noted. Future studies must perform another sampling procedure in multiple cities to improve the generalizability of our findings. At the same time, to reduce bias and increase the generalizability of the results, the sample size was considered as high as possible to enable more HCWs to participate as representatives of the target population. Furthermore, since the study’s target population was limited to employees in the healthcare sector, we tried as much as possible to sample all occupational groups involved in addressing COVID-19.

Despite the limitations mentioned above, the present study has obtained valuable information about the psychosocial status of the participating Iranian HCWs, which emphasizes their main concerns at the peak of the COVID-19 epidemic (the fifth wave) in Iran. This information can be used to implement appropriate interventions to reduce the effects of mental distress caused by COVID-19 or even other health-related crises in HCWs. The results offer valuable data for policymakers and mental health experts in general about the psychosocial influence of an outbreak of an infectious disease, which may help them in preparing for probable upcoming crises of various infections.

## Conclusion

The findings of the present study reveal the high rate of psychosocial distress among Iranian HCWs during the COVID-19 pandemic and its association with the perceived individual risk of infection. Thus, decreasing risk perceptions may improve HCWs’ physical and psychosocial health. This might be achieved by implementing regular PPE assessments to ensure its availability and quality, developing comprehensive mental health support programs for HCWs, and promoting workplace initiatives to reduce stigma and promote psychological wellbeing. Promoting the mental health of HCWs can improve the quality of patient care and the overall functioning of the healthcare system by increasing their job satisfaction. This, in turn, can cause an increase in their sense of usefulness; expand their resilience; improve their flexibility; deepen their interest in their job responsibilities; reduce anxiety, despair, and hopelessness; decrease human error in patient care; and improve the quality of their work and communication with patients. Consequently, the quality of care services provided to patients will improve.

As the psychosocial state among HCWs is a key subject related to the quality of services provided to patients infected with COVID‐19, health managers and officials of the MOH are recommended to offer psychosocial assessments and helpful care plans for HCWs with the purpose of improving their psychological health and skills for coping with serious situations. The responsibilities and actions that managers and policymakers should take to support HCWs’ psychosocial health could include providing resources and training for managers to address psychosocial concerns, ensuring the availability of mental health services and promoting a culture of support and wellbeing within healthcare organizations.

Follow-up investigations of different peaks of the COVID-19 crisis, or after it, might disclose the influences of COVID-19 on HCWs’ mental health. This research can be used to evaluate the effectiveness of measures targeted at promoting mental health and work-related wellbeing. Conducting a qualitative study and interviewing HCWs might generally clarify the nature and scope of the psychosocial influence of the COVID-19 crisis on employees in the health system. Additionally, other areas of future research that can build upon the current findings include, for example, exploring the long-term impacts of the COVID-19 pandemic on HCWs’ mental health or investigating the effectiveness of specific interventions or support programs. It also seems necessary to conduct longitudinal studies to track HCWs’ psychosocial health symptoms and design evidence-based interventions.

### Practical implications for healthcare policymakers, administrators, and mental health professionals

1. The results of this investigation hold significant implications for the enhancement of mental health policies and practices to support healthcare workers. This study can also contribute to the body of research that informs policymaking and practical measures aimed at preparing health systems for future pandemics.
2. The data presented herein may prove advantageous to researchers, stakeholders, and policymakers in their efforts to formulate sustainable, evidence-based interventions and guidelines to prevent or mitigate the immediate and long-term effects of infectious disease outbreaks on the mental health status of all HCWs.
3. Additionally, the knowledge gaps highlighted by this study may inform a forthcoming study concentrating on randomized controlled trials to assess the efficacy of various interventions while taking into account the motivators and obstacles influencing these interventions, in various economic, cultural, and social settings, to address the psychosocial problems of HCWs during infectious disease pandemics.

### Recommendations and strategies to reduce the psychosocial challenges confronting healthcare workers

The psychological effects on HCWs resulting from outbreaks and the aftermath are intricate and necessitate concerted efforts by governments and healthcare systems to address them in a sustained manner. It is essential to devise and execute intervention strategies that are interdisciplinary and collaborative in nature to alleviate these effects. Such strategies must be multifaceted and should be implemented, as our study recommends, at various levels, ranging from the intrapersonal level to public health.

At the individual level, self-help approaches are currently being implemented and have the potential to alleviate several ranges of psychosocial problems. It is imperative that HCWs receive proper expressions of gratitude due to the challenging work they undertake, as this can boost their resilience. Additionally, this acknowledgment should encompass awareness of possible psychological challenges and impart knowledge regarding accessible support resources.

At the interpersonal level, most of the HCWs deemed informal peer support tremendously helpful in surmounting arduous working circumstances. Various interventions implemented at both the individual and team levels, including education and training initiatives, targeted mental health interventions, and peer and social support, have the potential to mitigate negative mental health outcomes.

At the organizational level, it is imperative for managers to address several crucial factors. These include, but are not limited to, ensuring that adequate equipment is provided, implementing effective on-site infection control policies, and disseminating timely and accurate information. It is also important to make necessary adjustments to working hours, such as setting reasonable timelines and workloads, offering flexible work-from-home options, facilitating adequate rest, and providing compensation or flextime options to employees who perform overtime work. Additionally, managers can implement various measures to enhance the morale of HCWs and increase their self-confidence in situations where reducing working hours during pandemics is not an option. These measures can include planning to decrease the times of work shifts and workload, implementing rotational shifts for HCWs working in high-risk areas, employing new HCWs, offering well-timed breaks, inviting mental health specialists to provide advice, and beginning counseling meetings with easy access to the counselor.

At the community level, it is imperative to address any prejudice, stigma, or unfair behavior directed toward frontline HCWs participating in the pandemic response to safeguard the mental welfare of the workforce. Engaging in anti-stigma initiatives could prove to be efficacious in mitigating the pervasive sentiments of isolation and weariness that have been frequently reported in the aftermath of the initial outbreak of the pandemic.

At the level of public policy, the provision of targeted mental health support and the enhancement of working conditions may mitigate various psychological issues. As traumatic stress is characterized by avoidance behavior, which can be demonstrated through distressed personnel refraining from attending work, it is crucial to contact absent staff to determine whether their absence is linked to mental health issues.

Although our study has presented significant, novel findings, we must emphasize the pressing requirement for evidence-based procedures and policy recommendations that effectively avert and reduce the psychological consequences of COVID-19 on HCWs. This is particularly crucial because they will continue responding to forthcoming surges of this and future pandemics.

### Clinical implications

- Throughout the 1.5-year period of Iran’s severe COVID-19 outbreak, fairly high rates of PTS signs, psychosocial distress, and mental health problems were suffered by HCWs who had been at high risk of contracting COVID-19.

Acceptance and understanding of job-related risks may have protected some HCWs against negative psychosocial consequences of the COVID-19 crisis.

## Data Availability

The data collection scales and datasets created and/or analyzed through the present study are available from the corresponding author on reasonable request.

## Abbreviations

CE: Community engagement
NGOs: Non-governmental organizations
SI: Social innovation
SIH: Social innovation in health
WHO: World Health Organization
